# *Clostridium perfringens* necrotizing pancreatitis: an unusual pathogen in pancreatic necrosis infection

**DOI:** 10.1099/acmi.0.000261

**Published:** 2021-09-15

**Authors:** Ana Sánchez-Gollarte, Laura Jiménez-Álvarez, Marina Pérez-González, Cristina Vera-Mansilla, Alma Blázquez-Martín, Manuel Díez-Alonso

**Affiliations:** ^1^​ Department of General and Visceral Surgery, Hospital Universitario Príncipe de Asturias, Spain

**Keywords:** acute pancreatitis, *Clostridium perfringens*, clostridial pancreatic necrosis infection, pancreatic gas gangrene

## Abstract

Pancreatic necrosis infection (PNI) accounts for about 20–40 % of severe acute pancreatitis. PNI caused by anaerobic bacteria is unusual but when they present, *Clostridium perfringens* is the microorganism most commonly involved. We present a 60-year-old patient with a previous history of SARS-CoV-2, diagnosed with acute pancreatitis. During the hospitalisation he developed *Clostridium perfringens* bacteraemia. A CT-scan showed pancreatic gas gangrene and a surgical necrosectomy was performed. *Clostridium perfringens* was isolated in cultures of the pancreatic tissue and collections. The patient’s clinical status improved after surgery and the appropriate medical therapy. He was discharged 76 days after admission. Nowadays, the ‘step-up approach’ is an accepted therapeutic tool in treatment of pancreatic necrosis and peripancreatic fluid collections. However, most authors suggest that *Clostridum perfringens* infection requires a more aggressive approach due to the high mortality associated to clostridial infection.

## Introduction

Acute pancreatitis occurs when the pancreas becomes inflamed. It is characterized by upper abdominal pain, usually radiating to the back. Acute pancreatitis usually takes a mild course in most patients. However, the severe form, defined as acute pancreatitis associated with organ failure lasting more than 48 h, comprises ~20–30 % of cases with hospital mortality rates of ~15 % [1]. Among patients with severe pancreatitis ~20–40 % develop infection of the pancreatic and peripancreatic necrosis, which is known as pancreatic necrosis infection (PNI). PNI is associated with torpid evolution and higher organ failure rates. Different micro-organisms have been found to cause PNI, but *Clostridium perfringens* is particularly relevant due to the morbidity and mortality associated with this pathogen. We present a case of a spontaneous PNI by *C. perfringens* successfully treated with surgical necrosectomy.

## Case report

A 60-year-old patient with history of hypertension, diabetes and previous severe acute respiratory syndrome coronavirus 2 (SARS-CoV-2) infection (6 months earlier) was admitted to hospital with abdominal pain. Physical exploration revealed upper abdominal tenderness without signs of peritoneal irritation. Blood tests showed leucocytosis 15300 µl^−1^, glucose 168 mg dl^−1^, creatinine 1.35 mg dl^−1^, LDH 212 U l^−1^, GPT 14 U l^−1^, Na^+^144 mmol l^−1^, K^+^3.1 mmol l,^−1^ amylase 4530 U l^−1^ and lipase 2220 U l^−1^. A SARS-CoV-2 PCR test was negative. The antibody test was negative for IgM, while it remained positive for IgG.

**Fig. 1.**
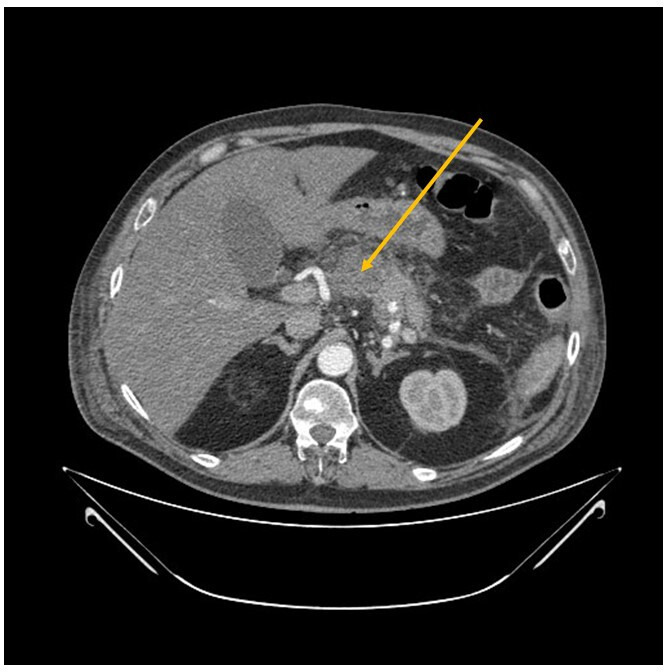
Forty-eight hour CT scan showing acute pancreatitis and peripancreatic fluid with no hypodense areas of pancreatic necrosis (yellow arrow).

**Fig. 2.**
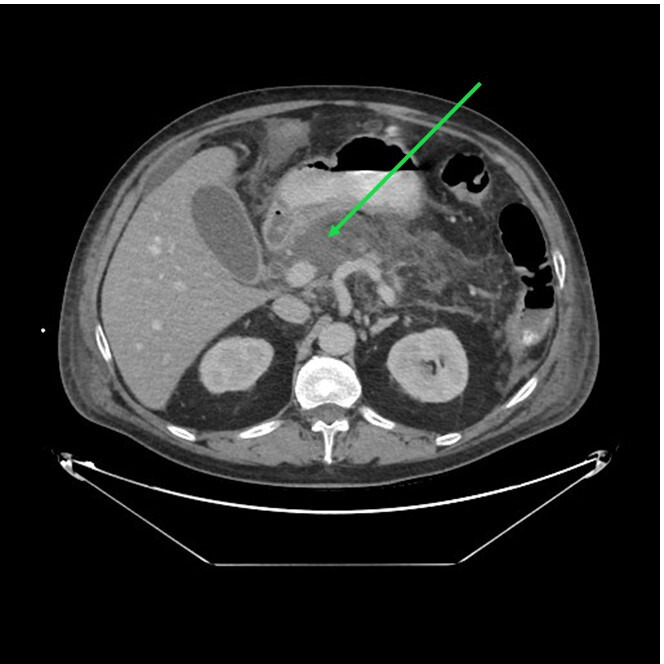
CT scan revealing hypodense areas corresponding to pancreatic necrosis (green arrow).

A diagnosis of acute pancreatitis was made with Ranson’s criteria. The patient was started on medical treatment based on fluid therapy and analgesia, but in the first 48 h his clinical status worsened progressively, presenting with four Ranson’s criteria for severity. The Ca^2+^ level was 7.1 mg dl^−1^, base deficit 7.6 mmol l^−1^, blood urea nitrogen was 38.6 mg dl^−1^ and fluid requirements were >6000 ml (Table 1). Computerized tomography (CT) was then performed, which found acute pancreatitis without necrosis, but accompanied with peripancreatic fluid collection (Fig. 1). With these clinical and radiological findings, the patient was admitted to the intensive care unit (ICU) for close surveillance and treatment optimization. During the first 5 days of ICU admission, the patient presented with fever peaks (reaching 39.3 °C), a rise of acute phase reactants and metabolic acidosis. Moreover, he needed high-flow oxygen and parenteral nutrition as he was kept nothing per mouth (NPO). A second CT scan was performed, which revealed necrotizing pancreatitis affecting up to the 40 % of the gland and there was also an increase of the peripancreatic fluid volume in relation to the previous CT scan (Fig. 2). Conservative treatments based on fluid, analgesic and oxygen requirements were intensified and 10 days later the patient was discharged to a conventional hospital ward.

**Table 1.** Ranson’s criteria at 0 and 48 h

Patient parameters according to Ranson’s criteria	
**0 h**	
Age	60
Leukocytes	15.300 µl^−1^
Glucose	168 mg dl^−1^
LDH	212 U l^−1^
AST	14 U l^−1^
**48 h**	
Haematocrit	43%
BUN	38.6 mg dl^−1^
Serum calcium	7.1 mg dl^−1^
pO_2_	95 mmHg
Base deficit	7.6 mmol l^−1^
Fluid needs	6000 l

**Fig. 3.**
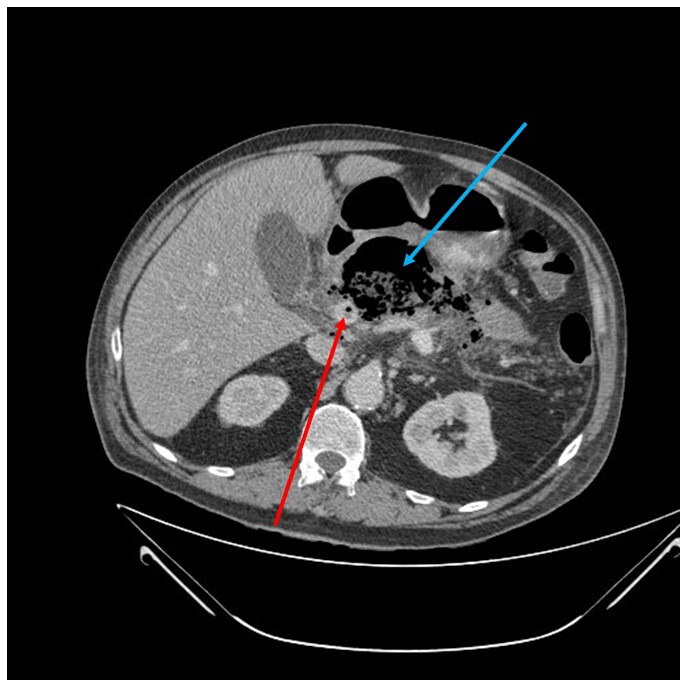
CT scan showing gas collection (blue arrow) and a thrombus inside the portal vein (red arrow). These findings characterize emphysematous pancreatitis and phylephlebitis.

Four days later (21 days after admission), the patient presented with persistent fever accompanied by rising leukocyte and C-reactive protein levels and worsening abdominal pain without any evidence of organ failure. In this context, broad-spectrum antibiotics (imipenem 1000 mg/6 h and vancomycin 1000 mg/12 h) were added to the treatment panel and blood cultures were also obtained, which were positive for *Clostridium perfringens *. A CT scan was then performed, which found emphysematous pancreatitis with gas and fluid collection and filling defect in the spleno-portal vein system and portal gas (Fig. 3). Surgical drainage of the collections was performed and cultures of the retroperitoneal abscess were also positive for *C. perfringens *. However, 72 h later, the patient remained febrile with no evidence of organ failure. A new CT scan revealed the persistence of peripancreatic collection, which was smaller than that detected by the previous CT. A second surgical intervention allowed the drainage of the remaining collections, and a cholecystectomy and pancreatic necrosectomy were also performed. The patient developed a pancreatic fistula as a postoperative complication and was treated conservatively with a drain. The anatomopathological analysis of the gallbladder revealed the presence of bile mud. After 76 days of hospitalization, the patient was discharged, with continued follow-up in the outpatient department.

## Discussion

Acute pancreatitis is an inflammatory condition of the pancreas that is most commonly caused by bile stones or excessive use of alcohol. During the current global coronavirus disease 2019 (COVID-19) pandemic, some cases of acute pancreatitis in COVID-19 patients have been reported in the literature [2, 3]. However, gallstone remains the main cause of acute pancreatitis and, although viral pancreatitis has previously been well described in the literature, the role of SARS-CoV-2 virus remains uncertain [4].

Pancreatic and peripancreatic necrosis can become infected by different bacteria, causing pancreatic necrosis infection (PNI) [1]. The micro-organisms usually involved are *Escherichia coli, Enterococcus* spp.*, Streptococcus* spp.*, Klebsiella* spp. *and Enterobacter* spp.*,* although polymicrobial infections are not rare. Even though infections by anaerobes are not very frequent, *C. perfringens* remains the principal agent involved, and is associated with an increasing mortality rate [5].

*C. perfringens* is a Gram-positive, sporeforming and strictly anaerobic bacillus that is frequently isolated from the biliary tree and gastrointestinal tract [5–7]. *C. perfringens* forms a part of the human normal intestinal and biliary flora of humans, but it can attain pathogenic potential in special scenarios such as anoxia, immunosuppression or in the presence of necrotic tissue [8]. In fact, older age and multiple comorbidities have been described as risk factors for acquiring *Clostridium* bacteraemia [6, 9]. In this particular case, diabetes could be the risk factor that predisposed the patient to clostridial bacteraemia and necrotizing pancreatic gas gangrene. It is well known that *C. perfringens* type A produces the major alpha-toxin, which is able to degrade cell membranes directly, leading to tissue necrosis [10].

The way in which *C. perfringens* infects pancreatic tissue is not well known. It is supposed that it reaches the gland either by retrograde duodenal infection, migration through the biliary tree, or by bacterial mural translocation from the colon due to increasing permeability of the colonic serosa in the context of inflamed pancreas tissue [8, 11–14]. However, spontaneous clostridial necrotic infection of the pancreas in the absence of enteric fistula or history of endoscopic or surgical procedures is extremely rare [8]. Similarly to the present case, Chirs *et al.* [11] reported two cases of pneumoretroperitoneum in patients with no history of previous biliary tree manipulation in which *C. perfringens* was isolated in surgical cultures of pancreatic tissue [11].

Clostridial pancreatic infection is a diagnostic challenge and requires a high index of suspicion. The presence of retropneumoperitoneum is usually related to perforation of hollow viscera, enteric fistula or anaerobic infection. In an acute pancreatitis patient with no previous history of bowel fistula or endoscopic procedures, pancreatic gas gangrene must be suspected when an abdominal CT scan shows the presence of gas in the pancreatic tissue or peripancreatic collections [15, 16].

The current treatment for pancreatic necrosis infection is known as the ‘step-up approach’. The first step is the percutaneous drainage of fluid collections, followed by endoscopic or minimally invasive surgical drainage of collections and resection of pancreatic necrosis if necessary [1]. There is little experience in the management of *C. perfringens* pancreatic necrosis infection, as only a few cases have been reported in the literature. Nevertheless, clostridial pancreatic infection quickly evolves towards multiple organ failure and is associated with a high morbidity and mortality rate, reaching ~80 % in the absence of early commencement of adequate supportive treatment [12]. In *C. perfringens* infection, organ failure may initially be absent. However, the worsening of acute phase reactants and the lack of improvement despite a correct resuscitation must make clinicians think of an atypical bacterial infection. Due to the severe consequences of this entity, the management of *C. perfringens* pancreatic necrosis infection may differ from the step-up approach currently recommended for the treatment of pancreatic necrosis. As in this patient, cases reported by Castro *et al.* and Chris *et al.* was successfully treated with surgical debridement [5, 11]. In that sense, most authors recommend an emergency surgical approach based on the drainage of collections and a wide debridement of pancreatic necrosis associated with intensive resuscitation and antibiotic therapy [5, 15–17].
